# Orbital tomography: Molecular band maps, momentum maps and the imaging of real space orbitals of adsorbed molecules

**DOI:** 10.1016/j.elspec.2015.04.023

**Published:** 2015-10-01

**Authors:** Hannes Offenbacher, Daniel Lüftner, Thomas Ules, Eva Maria Reinisch, Georg Koller, Peter Puschnig, Michael G. Ramsey

**Affiliations:** Institute of Physics, University of Graz, Universitätsplatz 5, 8010 Graz, Austria

**Keywords:** Photoelectron spectroscopy, Orbital tomography, Al(1 1 0), Pentacene, Sexiphenyl

## Abstract

The frontier orbitals of molecules are the prime determinants of their chemical, optical and electronic properties. Arguably, the most direct method of addressing the (filled) frontier orbitals is ultra-violet photoemission spectroscopy (UPS). Although UPS is a mature technique from the early 1970s on, the angular distribution of the photoemitted electrons was thought to be too complex to be analysed *quantitatively*. Recently angle resolved UPS (ARUPS) work on conjugated molecules both, in ordered thick films and chemisorbed monolayers, has shown that the angular (momentum) distribution of the photocurrent from orbital emissions can be simply understood. The approach, based on the assumption of a plane wave final state is becoming known as orbital tomography. Here we will demonstrate, with selected examples of pentacene (5A) and sexiphenyl (6P), the potential of orbital tomography. First it will be shown how the full angular distribution of the photocurrent (momentum map) from a specific orbital is related to the real space orbital by a Fourier transform. Examples of the reconstruction of 5A orbitals will be given and the procedure for recovering the lost phase information will be outlined. We then move to examples of sexiphenyl where we interrogate the original band maps of thick sexiphenyl in the light of our understanding of orbital tomography that has developed since then. With comparison to theoretical simulations of the molecular band maps, the molecular conformation and orientation will be concluded. New results for the sexiphenyl monolayer on Al(1 1 0) will then be presented. From the band maps it will be concluded that the molecule is planarised and adopts a tilted geometry. Finally the momentum maps down to HOMO-11 will be analysed and real space orbitals reconstructed.

## Highlights

• Orbital tomography within the plane wave final state approximation.
• One electron orbital predictions versus angle resolved photoemission experiment.
• Geometric and electronic structure of organic thin films elucidated by ARUPS.
• Influence of molecular conformation and orientation on ARUPS.
• Retrieval of sexiphenyl and pentacene orbitals in real space.

The frontier orbitals of molecules are the prime determinants of their chemical, optical and electronic properties. Arguably, the most direct method of addressing the (filled) frontier orbitals is ultra-violet photoemission spectroscopy (UPS). For instance, in the field of organic electronics, standard UPS is often used for studying energy level alignment and charge injection barriers of organic films. Organic molecules have a high propensity to form oriented and ordered structures. This leads to their photoemission having a very distinct angular distribution and the UPS spectra being extremely dependent on the experimental geometry and molecular orientation. If, as is often the case, this is ignored UPS can be misleading as, for instance, the frontier orbitals often have no emission intensity in standard experimental geometries. However, if it is understood angle resolved UPS (ARUPS or ARPES) can be an extremely powerful tool for studying both the geometric and electronic structure of molecular films and their interfaces [1], [2], [3], [4], [5], [6], [7], [8], [9], [10], [11], [12], [13], [14], [15], [16], [17], [18], [19], [20], [21], [22], [23], [24]. Prof. Ueno and his group at Chiba University were one of the first groups to highlight the importance of considering the full hemisphere of photoelectron emission to understand the electronic structure of device relevant conjugated molecules [12], [13], [14], [15], [16].

Although UPS is a mature technique, the angular distribution of the photoemitted electrons is generally thought to be too complex to be analysed *quantitatively*. The problem is how to treat the final state. In the early 1970s Gadzuk [25] proposed treating it as a single plane wave which leads to the simple result that the photoemission intensity distribution would be proportional to the Fourier transform (FT) of the initial state. A number of results in the mid 1970s, however, led to it being considered inadequate for the low emission energies of UPS [26]. With the plane wave approximation considered too simple the independent atomic center approximation (IAC) was then adopted. The IAC approximation calculates the emission as an independent, but coherent, sum of spherical waves from individual atomic centres. The IAC can be further developed by considering scattering of the photoemitted electrons. Ueno and his group have been applying the IAC to interpret the angular dependence of their photoemission results from large molecules [12], [13], [14], [15], [16].

More recently it was found that the band maps of thick crystalline films of linear molecules could be well described by the FT of the initial state orbitals making up the molecular bands [1], [2], [3], [4], [5], suggesting that the plane wave approximation was a useful description. These, essentially 1 dimensional examples inspired work on 2 dimensional systems where it was found that the momentum distribution for a variety of adsorbate monolayers also agreed with expectations from the FT of the initial state wave functions [3], [4], [5], [6], [7], [8], [9], [10], [11]. In the last few years it has been demonstrated that by slicing up the photoemission data cube (photocurrent *I*(*E*, *k*_*x*_, *k*_*y*_)), the thus generated band maps (*I*(*E*, *k*)) and momentum maps (*I*(*k*_*x*_, *k*_*y*_) at constant binding energy) can usefully be interpreted within the plane wave approximation. This is becoming known as orbital tomography which allows one to determine molecular geometries [3], [11], gain insight into the nature of the surface chemical bond [7], [17], [18], unambiguously determine the orbital energy ordering in molecular homo- and heterostructures [8], [9], [18] and even reconstruct orbitals in real space [3], [10]. All these results indicate that a simple Fourier transform of the initial state wave functions describes the photoelectron distribution and imply that a plane wave is a good approximation for the final state in photoemission [3]. It is worth noting here that the independent atomic centre approximation (IAC) can be shown to reduce to the plane wave final state result [3], [27] if;

• all contributing atomic orbitals are of the same type (e.g. p_z_-orbitals),
• the electron emission direction is close to the polarisation vector of the incoming photon,
• the molecule consists of only light atoms (C, N, O) with small scattering cross sections.

To a certain extent most of the orbital tomography experiments on the orbitals of conjugated molecules till now do indeed come close to satisfying these conditions.

In the following we will demonstrate, with selected examples of pentacene (5A) and sexiphenyl (6P), the potential of orbital tomography. First it will be shown how the full angular distribution of the photocurrent (momentum map) from a specific orbital is related to the real space orbital by a Fourier transform. Examples of the reconstruction of 5A orbitals will be given and the procedure for recovering the lost phase information will be outlined. We then move to examples of sexiphenyl. Here we will interrogate the original band maps of thick sexiphenyl [1] in the light of our understanding of orbital tomography that has developed since then. With comparison to theoretical simulations of the molecular band maps, the molecular conformation and orientation will be concluded. New results for the sexiphenyl monolayer on Al(1 1 0) will then be presented. From the band maps it will be concluded that the molecule is planarised and adopts a tilted geometry. Finally the momentum maps down to HOMO-11 will be analysed and real space orbitals reconstructed.

The experiments (base pressure < 5 × 10^−10^ mbar) have been performed on the toroidal energy analyser at the synchrotron radiation facility BESSY II of the Helmholtz-Center Berlin (HZB) (see Refs. [3], [7]). This spectrometer is unique in that it can collect the full hemisphere of emitted electrons without changing the photon incident angle (polarisation). Moreover, there is no restriction on the photon incident angle. The Al(1 1 0) substrate was judged clean after sputter and annealing cycles if ARUPS of the valence band region only showed the weak Al sp-band (i.e. no contaminant emissions) and a work function of 4.3 eV. The simulated band maps were obtained via Fourier transformed orbitals resulting from ab-initio density functional theory (DFT). Details of the calculations can be found in the comprehensive theoretical investigation of phenyl and acene oligomers of Ref. [28].

**Fig. 1:**
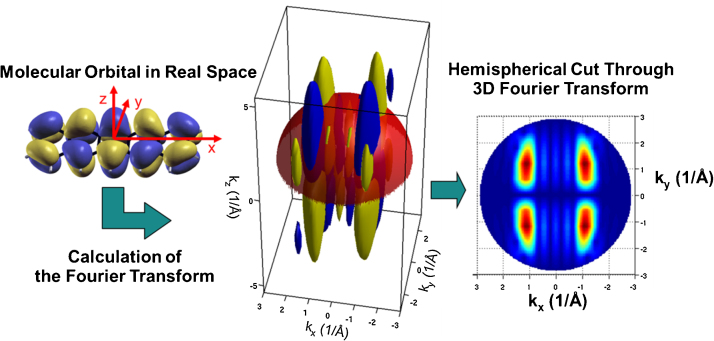
Relationship between the real space molecular orbital and the photoemission momentum map. At a particular photon energy the momentum *k* of the photoemitted electron is $k=\sqrt{\left(2m/h^{2}\right)E_{kin}}$ as indicated by the red hemisphere. The absolute value of the orbitals Fourier Transform on this hemisphere yields the momentum map [3].

The relationship between the measured photoemission intensity and the FT of the emitting orbital can be illustrated with an Ewald sphere like construction. Fig. 1 shows the HOMO of pentacene (5A) in real space and its corresponding three-dimensional FT, the orbital in reciprocal space. The colour represents the sign or phase of the wave function. The photoemission from the orbital will lead to a particular kinetic energy and *k* value, represented by the red hemisphere. The intersection of this hemisphere with the orbital in reciprocal space gives the coordinates for the emission from this orbital. The value of the FT on the hemisphere for a kinetic energy of 29.8 eV is shown in Fig. 1. The square of this hemispherical cut yields the prediction for the experimental momentum map for a flat lying 5A molecule.

**Fig. 2:**
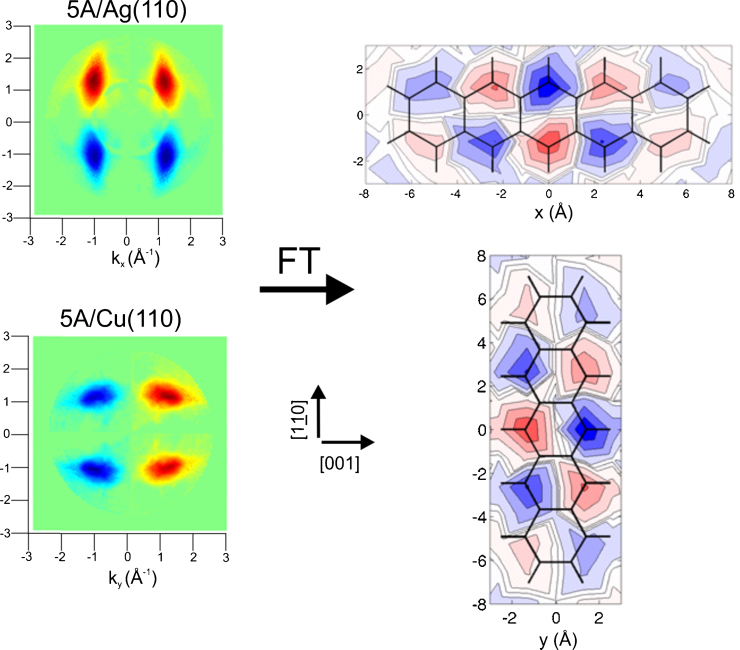
Momentum maps of the HOMO of pentacene from monolayers adsorbed on Ag(1 1 0) and Cu(1 1 0) taken at binding energies of 1.2 and 1.5 eV, respectively, using a photon energy of 35 eV and an angle of incidence of 40°. The sign (red and blue) of the wavefunctions has been assumed and imposed on the data and the result of the Fourier transforms are in good agreement to the real space orbital.

The first back FT from measured momentum maps to real space orbitals were performed for the HOMO and LUMO orbitals (the latter occupied on bonding) of sexiphenyl adsorbed on Cu(1 1 0) [3]. For this the phase, which is naturally lost in experiment, was imposed onto the momentum map data. This procedure is illustrated in Fig. 2 for data from the HOMO of pentacene adsorbed on both Ag(1 1 0) and Cu(1 1 0) surfaces. From the momentum maps one sees immediately that the molecule adopts different azimuthal orientations on the two surfaces; on Ag it aligns along the [0 0 1] while on Cu it is aligned parallel to the close packed row in the [1], [2], [3], [4], [5], [6], [7], [8], [9], [10] direction. Comparison to the theoretical real space orbital and predicted momentum map of Fig. 1 also indicates that the molecules have their aromatic plane parallel to the surface. Moreover, the results suggest that the HOMO orbitals of the adsorbed molecules on both surfaces are essentially unaltered from that of the free molecule. This is somewhat surprising as in the case for 5A on Cu(1 1 0) the molecule shows strong hybridisation with the surface with the LUMO expressing a large substrate induced intermolecular dispersion and a momentum map that indicates a lateral expansion of 20%. A detailed comparison of 5A on Ag and Cu may be found in Ref. [29]. It is also worth noting that if scattering was to be a major problem to the plane wave approximation it should have manifested itself in the maps of Fig. 2 due to the differences of orientation on Ag and Cu.

**Fig. 3:**
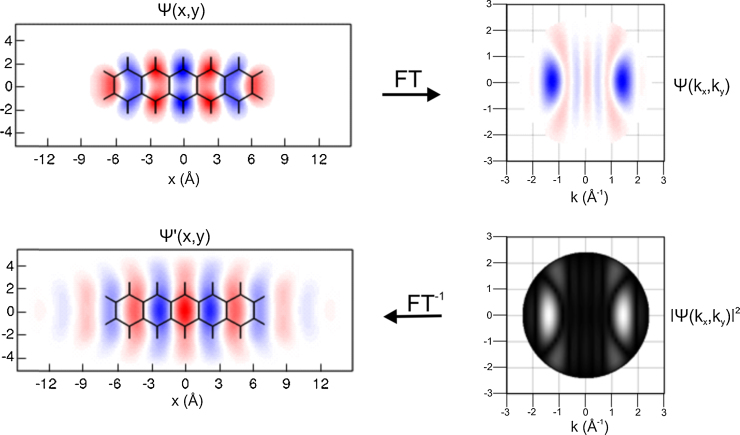
Illustration of the phase problem for the LUMO of pentacene. Top: The real space DFT orbital (10% isosurface) and the Fourier transform of the orbital at *k* = 2.5 Å^−1^. The phase of the wavefunction is indicated in colour. Bottom: The resulting simulation of an experimental momentum map with the loss of phase and the result of the transformation back to real space.

**Fig. 4:**
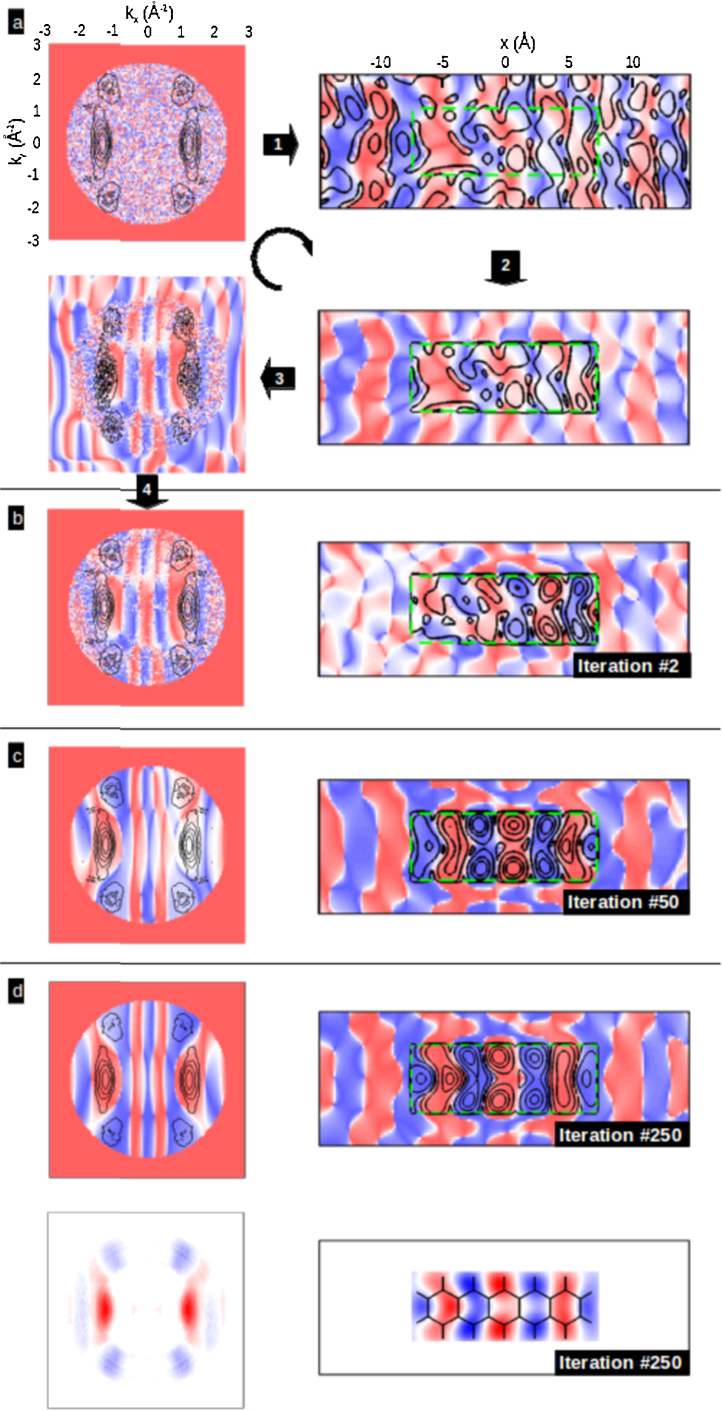
Iterative phase recovery and real space orbital reconstruction of the LUMO of pentacene on Ag(1 1 0). (a) First loop in the iteration starting with the momentum map measured at 0.15 eV binding energy. Black isolines represent the square root of the photoemission intensity and the colours represent the sign of the wavefunction starting with random phase. The steps 1 to 4 indicate the FT to real space, imposition of the confinement box (green rectangle), FT back to reciprocal space and imposition of the new phase on the original experimental map, respectively. The results after (b) 2, (c) 50 and (d) 250 iterations are shown.

Unlike the case for the 5A HOMO of Fig. 2 or the LUMO and HOMO of 6P [3], for many orbitals assuming the phase in the momentum maps is a priori not possible. An example of this would be the LUMO of 5A whose calculated real space orbital and simulated momentum map is shown in Fig. 3. This figure illustrates the phase problem where it is seen that without phase information the FT of the momentum map will lead to a real space orbital of twice the size with an incorrect phase. Recently we have shown how the phase information lost in photoemission can be objectively retrieved by applying an iterative oversampling procedure to the ARUPS momentum maps with the only assumption being the spatial confinement of the orbitals [10]. The procedure is illustrated for the case of the LUMO of 5A which is half filled and appears at the Fermi level when adsorbed on Ag(1 1 0). One starts with the experimental momentum map, expressed in Fig. 4 by the black isolines of the square root of the ARUPS intensity, with an arbitrary phase imposed, in this case a random phase. The FT of step 1 leads to a wave function randomly distributed over real space. The not unreasonable assumption that the wave function should be confined to the molecule is then made. This is done by creating a simple rectangular confinement box with roughly the Van der Waals dimensions of 5A and reducing the intensity outside the box by 90% in step 2. The inverse FT (step 3) then leads to a new phase (colour map) and intensity distribution (isolines) in momentum space. The new phase is then applied to the original ARUPS intensity in step 4 to close the iterative loop. As the procedure is repeated the probability distribution inside the confinement box increases at each iteration and the orbital becomes recognisable. The procedure is stopped when no further changes occur (here 250th iteration) and the result is then displayed as the real part of the wave function in real and momentum space, respectively, as a density plot. The resultant orbital is in very good agreement with the theoretical orbital of the isolated molecule of Fig. 2. The reconstructed orbitals are interpreted as planar 2-dimensional sections through 3-dimensional orbitals. Such an interpretation is reasonable for the molecular systems and orbitals we have measured so far; π orbitals of molecules with planar adsorption geometries. For these the 3D structure of the orbital in *momentum* space is dominated by features with weak *k*_*z*_ dependence that is essentially equal to that of an atomic *p*_*z*_ state. Therefore, the hemispherical cut through 3D momentum space, as measured in ARUPS with a single photon energy, can be interpreted as a 2D cut through the real space orbital [10].

With angle resolved UPS (ARUPS) work on conjugated molecules in crystalline thick films [1], [2], [3], [4], [5], it was shown that the angular (momentum) distribution of the photocurrent along a specific azimuthal direction *E*(*k*), i.e. a so called band map, can be simply understood. With examples of intra- and inter-band dispersion in thick molecular films of the chain like molecules sexiphenyl (6P) [1], sexithiophene (6T) [4] and pentacene (5A) [2], [5] we first demonstrated that a simple one dimensional Fourier transform of molecular orbitals predicts the, hitherto apparently complex, angular/momentum distribution of the emitted photoelectrons. With the understanding of orbital tomography that has developed since this original work, we can now interrogate previous data to expose new details. Specifically here we will revisit the band map measured for a thick 6P(20-3) film grown on the oxygen reconstructed Cu(1 1 0) surface [1]. The 6P crystallite orientation is such that all molecules have their long molecular axis parallel to each other and the substrate surface. X-ray diffraction tells us that the molecules are on average planar with the molecular plane tilted at ±33° to the surface plane [30], [31].

**Fig. 5:**
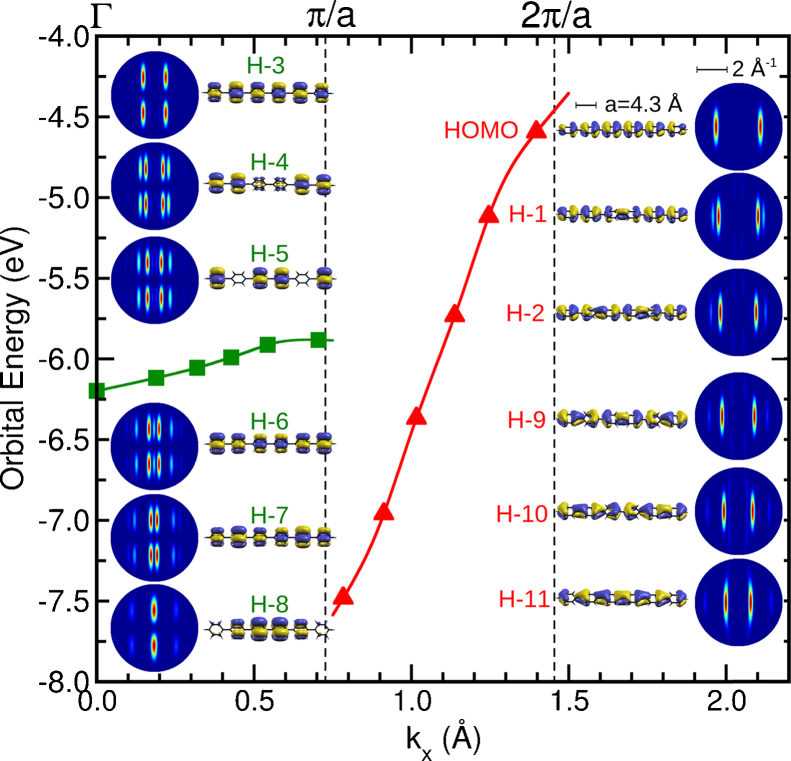
The π orbitals of planar sexiphenyl with their DFT-PBE energies plotted against the momentum associated with the principle periodicity in the direction of the long molecular axis, *k_x_*. For the inter-ring non-bonding orbitals (green) and inter-ring bonding orbitals (red) the momentum maps (*k_x_*, *k_y_*), simulated for a photon energy of 50 eV, are included. (For interpretation of the references to colour in this figure legend, the reader is referred to the web version of this article.)

As a reference, Fig. 5 shows the π orbitals of a planar 6P molecule as calculated with the DFT-PBE functional. The energy scale is with respect to the vacuum level and the *k*_*x*_ values indicate the principle periodicity of the orbitals along the long molecular axis. The Brillouin zone boundaries expected for an infinitely long phenyl chain, π/*a* and 2π/*a* where *a* is the inter-ring spacing, are indicated. The two upper π bands can be recognised as linear combinations of the two degenerate e_2*g*_ orbitals that make up the HOMO of benzene. The six orbitals of the green band (HOMO-3 to HOMO-8) have little energy dispersion and will be called the inter-ring non-bonding band. The red band (HOMO to HOMO-3 and HOMO-9 to HOMO-11) in contrast has significant dispersion as the “benzene” orbitals of neighbouring rings are spatially nearer each other, this band is consequently named the inter-ring bonding band.

**Fig. 6:**
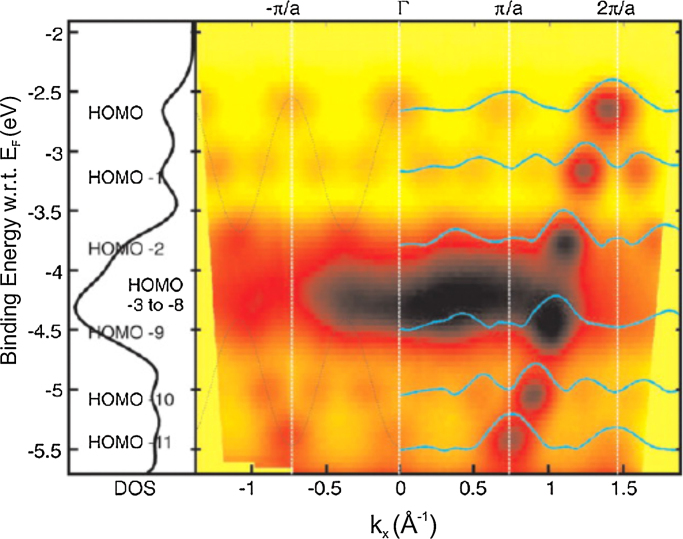
Band map of a thick 6P(20-3) oriented film using unpolarised He I excitation with an angle of incidence of 60°. The ARUPS data was collected in the plane of incidence, which contained the long molecular axis of the molecules. The binding energy is referenced to the Fermi level and the workfunction was measured to be 4.0 eV. The overlaid blue lines are the FT of the DFT orbitals of an isolated twisted sexiphenyl molecule. From Ref [1]. (For interpretation of the references to colour in this figure legend, the reader is referred to the web version of this article.)

A set of valence band photoemission spectra from the highest lying π orbitals was measured as a function of electron emission angle (*θ*) in the direction along the molecular axis. The orbital emissions exhibited distinct maxima at discrete *θ* values because different take-off angles probe different momenta. After converting *θ* to momentum,

$$k=\sqrt{\frac{2mE_{kin}}{h^{2}}}\;\text{sin}\;\theta$$

the π band map of sexiphenyl, *E*(*k*_*x*_), is obtained (Fig. 6). The partial density of states, derived from integrating the data over *k*_*x*_, is included on the side, and the orbitals of the bonding π band are labelled, starting with the HOMO.

The experimental band map shows a text book example of band structure formation. The 6 orbitals running down from gamma to the BZ boundary and up again in the 2nd zone to 2π/*a* are as expected for the inter-ring bonding band. The turning points/boundaries are not exactly at *n*π/*a* as the molecule is a nano structure and the periodicity of the electron density is slightly larger than the atomic backbone. Closer inspection also reveals band folding in that there are in fact two mini bands with 3 orbitals each. The boundaries are at ∼π/2*a* which can be attributed to the molecule being twisted leading to a periodicity of *2a* twice that of the planar molecule. The isolated molecule is calculated to have a torsional angle between rings of ∼35°, due to steric hinderance of the hydrogens, while X-ray diffraction studies had concluded the molecule in the crystal to be on average planar at room temperature [30].

At the time of the original work we were puzzled as to why the emissions of sexiphenyl and sexithiophene [4] were strong in the 2nd Brillioun zone, from π/*a* to 2π/*a*, while both π bands of pentacene were strongest in the 1st zone of the intermolecular band [2]. This puzzle was solved when the Fourier transforms of the calculated real space orbitals were taken. These were found to predict well the k-space positions and widths as well as the relative emission intensities. This is illustrated in Fig. 6 where the Fourier transforms of the orbitals of a *twisted* 6P molecule, projected on the *k*_*x*_ axis, is overlaid on the band map. The band folding induced by molecular twist is well expressed by, for instance, the appearance of features around π/*a* in HOMO and HOMO-1 and at 2π/*a* for HOMO-11.

The strong appearance of the band of non-bonding orbitals in *k*_*x*_ is another manifestation of tilting of the aromatic planes in the molecule. For the planar molecule the bonding orbitals have no nodal plane in *x*–*z*, where x is the long molecular axis and *z* is perpendicular to the aromatic plane. As a consequence they are observed in the *k*_*x*_ direction. The non-bonding orbitals have nodal planes in both *x*–*z* and *y*–*z* and thus should not be observed in the *k*_*x*_ direction. This is best expressed in the calculated momentum maps of the orbitals which are included in Fig. 5. One can imagine on tilting the aromatic plane the lobes of the non-bonding band orbitals in *k*-space will move in *k*_*y*_ such that they could cross the *k*_*x*_ axis and become observable in this direction. The effects of molecular tilt on momentum maps and the power of orbital tomography to determine the tilt in molecular multilayers and monolayers can be found in [3], [11].

**Fig. 7:**
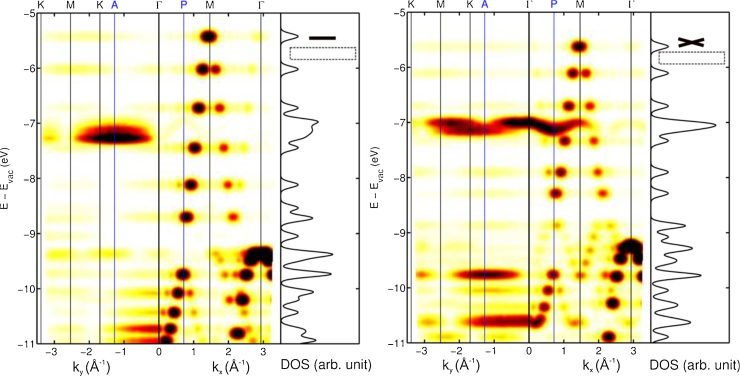
The simulated ARUPS band maps perpendicular (*k_y_*) and along the molecular axis (*k_x_*) for (a) planar and (b) 30° twisted sexiphenyl. The simulations were made using the Fourier transforms of the DFT-HSE wavefunctions broadened in energy by 100 meV and assuming a photon energy of 50 eV. For each the calculated density of states is shown on the right.

Higher level calculations using the HSE hybrid functional to ameliorate errors of self-interaction were also performed on both planar 6P and 6P with a torsional angle of 30°. Fig. 7 presents the results of the Fourier transforms of the wavefunctions in the *k*_*y*_ and *k*_*x*_ directions along with the density of states over all *k*. An energy broadening of 100 meV has been applied to simulate the experimental band maps. For the planar molecule, Fig. 7a, only the HOMO-8 is observed along *k*_*y*_ and none of the non-bonding band is predicted in the *k*_*x.*_ As predicted, on going to the twisted molecule the dispersing non-bonding band appears in *k*_*x*_ direction and the simulation, Fig. 7b, is in reasonable agreement with the experimental band map of Fig. 6.

**Fig. 8:**
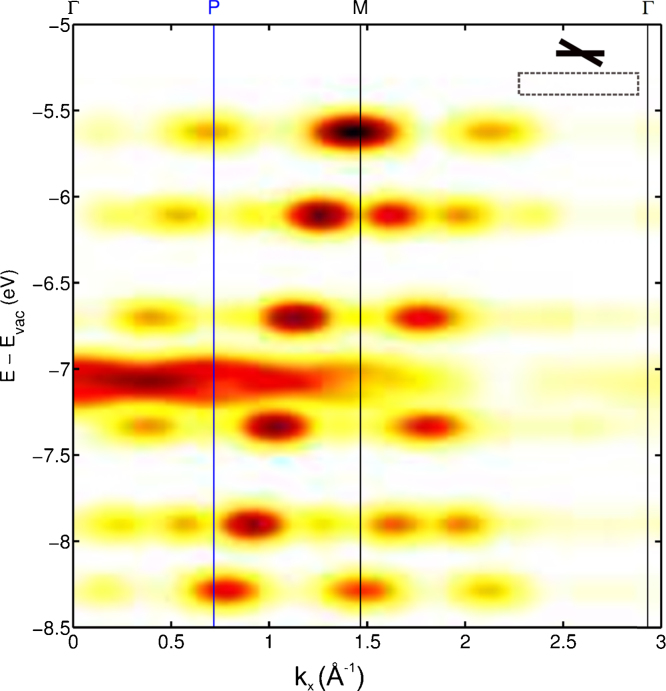
The simulated ARUPS band maps along the molecular axis (*k_x_*) for 30° twisted sexiphenyl that is also tilted by 15°, as indicated by the insert. Note the appearance of the features due to band folding absent in the untilted case (Fig. 7b.).

A third indicator for the molecules being in a twisted conformation is the energy spread of the bonding π band. In going to a twisted molecule the dispersion will decrease as the inter-ring orbital overlap is reduced. Indeed Fig. 7 shows the calculated HOMO to HOMO-11 separation going from 3.1 eV to 2.7 eV for a 30° torsional angle. Not only is the latter value identical to the band spread measured for the thick film, the band also shows an asymmetry in the band spread that is also observed in Fig. 6. This asymmetry is best expressed by the difference between HOMO and HOMO-1 energy gap relative to that between HOMO-10 and HOMO-11. For multilayer films they are 0.52 to 0.38 eV, respectively. These are very similar to the calculated separations in the 30° twisted molecule of 0.55 and 0.40 eV and quite different from those calculated for the planar molecule, which are almost identical at 0.57 and 0.55 eV. The simulated band map of Fig. 7b however does not reproduce the experimentally observed features of band folding. This can be understood as a twist of the molecule will hardly change the periodicity of the electron density along the axis of the molecule, but off the axis the double periodicity will be strongly expressed. This will lead to the *k*-space lobes, due to double periodicity, appearing at non-zero *k*_*y*_ in the momentum maps. To be observed in a *k*_*x*_ band map the molecule must also be tilted around its long axis. The theoretical *k*_*x*_ band map for a 6P molecule with a 30° torsional angle and a tilt of 15° is shown in Fig. 8. This simulation is in remarkably good agreement to the 6P(20-3) experiment of Fig. 6 expressing band folding, the appearance of the non-bonding band as well as the correct energy spread of the bonding π band.

Before leaving the HSE calculations it is worth commenting on the sigma orbitals and whether they can lead to misinterpretations. In the calculated density of states of the planar molecule of Fig. 7a three σ orbitals can be discerned around the energy of HOMO-11 (in standard PBE calculations without correction for self-interaction errors they are at even lower binding energies relative to the π orbitals and appear around the energy of HOMO-10). They, however, do not appear in the simulated band map as they have a much greater k values and will not appear in the experiment for photon energies below ∼50 eV [6]. Moreover, on twisting the molecule, the σ orbitals shift by ∼1 eV to higher binding energy relative to the π’s, and are no longer in the energy range of the upper π band.

**Fig. 9:**
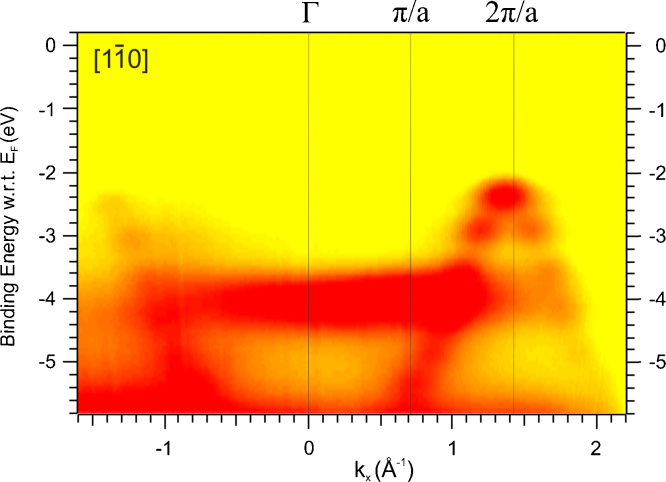
Band map of a 6P monolayer adsorbed on Al(1 1 0) generated by polarised 30 eV photons with an angle of incidence of 40°. The data was collected in the plane of incidence which contained the plane of polarisation and the [1], [2], [3], [4], [5], [6], [7], [8], [9], [10] azimuth of the substrate. The binding energy is referred the Fermi level and the workfunction was measured to be 3.9 eV.

We now turn to data for a monolayer of 6P adsorbed on Al(1 1 0). The band map in the [1], [2], [3], [4], [5], [6], [7], [8], [9], [10] substrate azimuthal direction in Fig. 9 is similar to that of the thick film of Fig. 6. The 6 orbitals of the bonding band clearly indicate that the long molecular axes of the molecules are aligned parallel to this substrate azimuth i.e. along the closed packed atomic rows of the surface. The band width, HOMO to HOMO-11 of 3.0 eV is, however, greater than the 2.7 eV measured for thick films [1], [7] and is close to the 3.1 eV of the calculations of Fig. 7a for a planar molecule. The asymmetry of the band spread is also significantly different to that of the multilayer film of Fig. 6. The difference in energy between HOMO and HOMO-1 and between HOMO-10 and HOMO-11 are almost identical at 0.53 and 0.5 eV and are very similar to the HSE calculated values of 0.57 to 0.55 eV for a planar molecule. Thus, both the results of band spread and band asymmetry suggest a planarisation of the molecule in the monolayer on Al. This is also supported by the lack of features of band folding. However, in both multilayer and monolayer on Al(1 1 0) the non-bonding orbitals appear strongly in the *E* vs. *k*_*x*_ band maps. While in the multilayer this can be attributed to a twisted molecule, for a planar molecule this can result from the molecules being tilted away from the surface around the long molecular axis.

The results of the monolayer on Al(1 1 0) can be compared and contrasted to the 6P monolayer on Cu(1 1 0) where the molecule is planarised and also oriented along the [1], [2], [3], [4], [5], [6], [7], [8], [9], [10] azimuth [7]. Unlike the monolayer result on Al, on Cu the non-bonding orbitals do not appear in the *E vs k*_*x*_ band maps indicating the aromatic planes are parallel to the surface. The Al result shows no evidence for an emission due to LUMO occupation. In contrast, on Cu(1 1 0) there is direct evidence for hybridisation with the LUMO observed below the Fermi level, 2 eV above the HOMO, with *k* = 1.5 Å^−1^. Finally the π band spread on Cu is 3.6 eV, considerably larger than that on Al and that calculated for the planar molecule. Although the planarisation of the molecule alone increases the π band width, on Cu, a greater contribution arises directly from mixing of molecular states with metal states [7]. The bond to the Al surface must thus be considered near purely van der Waals, nevertheless it is strong enough to planarise the molecule.

**Fig. 10:**
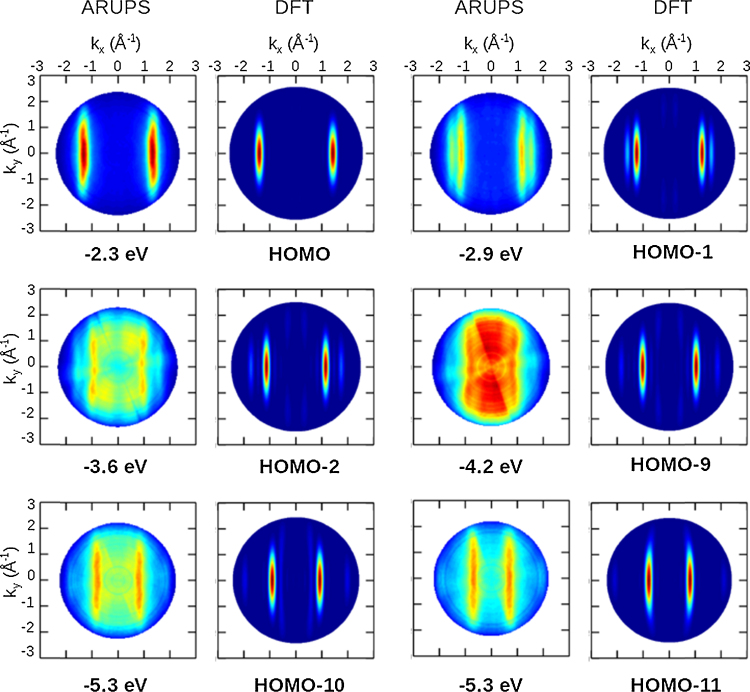
Momentum maps measured at the energies of the bonding π band emissions of Fig. 9 along with simulated maps for these orbitals.

The momentum maps taken at the energies of the emissions observed in the band map are shown in Fig. 10 along with the calculated *k*_*x*_, *k*_*y*_ maps for the orbitals of the bonding band of a flat lying planar molecule. These orbitals are characterised by lobes at specific *k*_*x*_ values that are elongated in *k*_*y*_. HOMO, HOMO-1, HOMO-10 and HOMO-11 can be immediately identified with the only difference in the experiment being that the lobes have a greater extension in the *k*_*y*_ direction. This extension can be attributed to a small tilt of the molecule around its long axis, away from the surface plane (on the surface an equal number of + and − tilts can be expected). HOMO-2 and HOMO-9 cannot be distinguished as they overlap with the six non-bonding orbitals. The calculated momentum maps of the non-bonding orbitals can be found in Fig. 5. These orbitals have lobes centred at *k*_*y*_ = ±0.8 Å^−1^ that are elongated in *k*_*y*_. Their *k*_*x*_ values range from zero for HOMO-8 to ±1 Å^−1^ for HOMO-3. At the HOMO-2 energy of 3.6 eV the experimental momentum map reveals features that can be recognised as those of HOMO-3 and HOMO-4. The momentum map at the HOMO-9 energy of −4.2 eV presents an intense rectangular emission that can be associated with the summation of all non-bonding orbitals, HOMO-3 to -8.

**Fig. 11:**
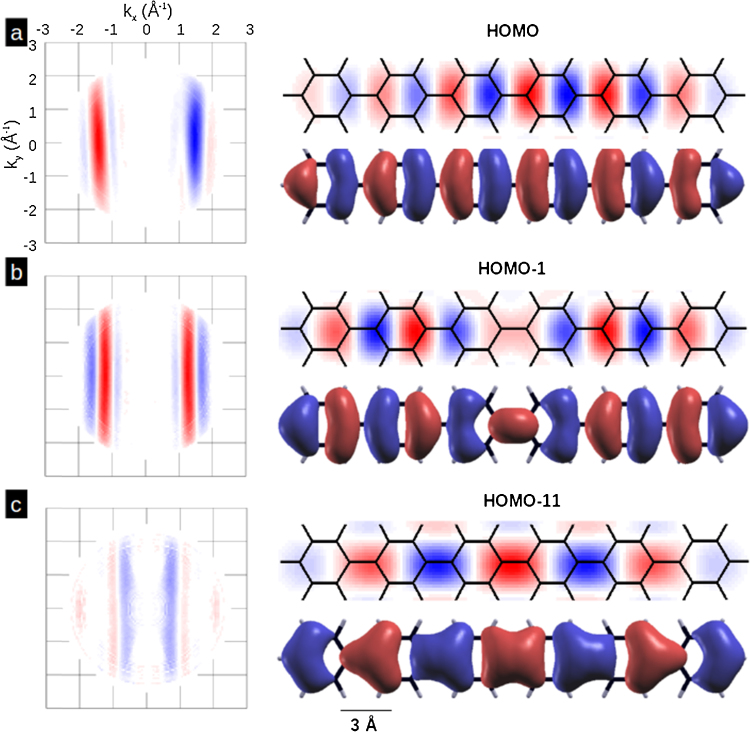
Results of the iterative phase recovery and orbital reconstruction from the momentum maps of the 6P monolayer on Al(1 1 0) of Fig. 10.

The ultimate expression of the power of orbital tomography and the viability of the plane wave final state approximation for UPS is the reconstruction of real space orbitals. The iterative phase recovery and orbital reconstruction procedure outlined above was applied to the HOMO, HOMO-1 and HOMO-11 momentum maps of Fig. 10 assuming a confinement box of 27.0 × 4.4 Å. The results of the experimental maps after phase recovery and the resulting real space orbitals are summarised in Fig. 11. Included in the figure are the calculated DFT orbitals of an isolated planar 6P molecule; top view with a 10% iso-surface i.e. a representation where 90% of the charge density is enclosed. The recovery of both the real space electron density distribution and the sign of the wave function goes well beyond merely identifying the character of the orbital emissions. For instance one sees that the HOMO is entirely inter-ring anti-bonding, HOMO-11 entirely inter-ring bonding while for HOMO-1 only the middle of the molecule is inter-ring bonding. While the experimentally obtained orbitals are in good agreement with those of theory the agreement is not perfect. The exact shape of the lobes do not entirely match the theory and the lateral extent of the lobes is slightly smaller. This can be attributed to the molecule not being entirely flat on the surface rather than to a change in the orbitals on adsorption. On a more general note, the good agreement of the experimentally determined orbitals to the one electron wave functions of ab initio calculations open up a window for discussion of the theoretical concept of orbitals. In the photoemission process one goes from N to an N-1 ionised final state and, theoretically, it should not reveal (one electron) molecular orbitals but rather (many electron) Dyson orbitals [10], [32]. How much the Dyson orbital differs from the molecular orbital will depend on the degree of electronic relaxation of the N-1 system on the creation of the photohole [32]. The results here would suggest that, even for the relatively deep lying HOMO-11, orbital the frozen orbitals approximation appears reasonable.

In conclusion, we have attempted a necessarily incomplete review of valence band orbital tomography. Examples of the interpretation of band and momentum maps within the plane wave approximation have been given to show it to be simple and predictive. As there is a considerable development in the area of electron spectrometers ongoing, we foresee orbital tomography being accessible to many more research groups. For instance, hemispherical analysers can now be purchased that allow angle resolved data to be obtained simultaneously over a range of 40° take off angle. Top of the range photoemission electron microscopes (PEEM) with energy filters can collect the full hemisphere of photoemitted electrons [33], while angle resolved time of flight spectrometers (ARTOFS) are being developed, which will allow high energy and angular resolved data from sensitive organic films, even when they are insulating. All these spectrometers can perform orbital tomography to a certain extent, however, with some constraints and complications regarding polarisation. For instance with both PEEM and ARTOF the condition of emission direction being close to the polarisation vector of the incoming photon is not met as they collect data in both specular and off-specular planes—what used to be referred to even and odd geometries [34]. We are presently performing experiments to explore the limits of the plane wave approximation. These involve systematic studies of the polarisation and energy dependence for both σ and π orbitals which we believe will make orbital tomography even more quantitative and more generally applicable.
